# Effects of hyperoxia on vascular tone in animal models: systematic review and meta-analysis

**DOI:** 10.1186/s13054-018-2123-9

**Published:** 2018-08-04

**Authors:** Bob Smit, Yvo M. Smulders, Etto C. Eringa, Heleen M. Oudemans - van Straaten, Armand R. J. Girbes, Kimberley E. Wever, Carlijn R. Hooijmans, Angelique M. E. Spoelstra - de Man

**Affiliations:** 1 Department of Intensive Care, VU University Medical Center, De Boelelaan 1117, 1007 MB Amsterdam, The Netherlands; 2 Department of Internal Medicine, VU University Medical Center, Amsterdam, The Netherlands; 3 Department of Physiology, VU University Medical Center, Amsterdam, The Netherlands; 4 SYstematic Review Centre for Laboratory animal Experimentation (SYRCLE), Department for Health Evidence, Radboud Institute for Health Sciences, Radboud University Medical Center, Nijmegen, The Netherlands

**Keywords:** Hyperoxia, Microcirculation, Vasoconstriction, Meta-analysis

## Abstract

**Background:**

Arterial hyperoxia may induce vasoconstriction and reduce cardiac output, which is particularly undesirable in patients who already have compromised perfusion of vital organs. Due to the inaccessibility of vital organs in humans, vasoconstrictive effects of hyperoxia have primarily been studied in animal models. However, the results of these studies vary substantially. Here, we investigate the variation in magnitude of the hyperoxia effect among studies and explore possible sources of heterogeneity, such as vascular region and animal species.

**Method:**

Pubmed and Embase were searched for eligible studies up to November 2017. In vivo and ex vivo animal studies reporting on vascular tone changes induced by local or systemic normobaric hyperoxia were included. Experiments with co-interventions (e.g. disease or endothelium removal) or studies focusing on lung, brain or fetal vasculature or the ductus arteriosus were not included. We extracted data pertaining to species, vascular region, blood vessel characteristics and method of hyperoxia induction. Overall effect sizes were estimated with a standardized mean difference (SMD) random effects model.

**Results:**

We identified a total of 60 studies, which reported data on 67 in vivo and 18 ex vivo experiments. In the in vivo studies, hyperoxia caused vasoconstriction with an SMD of − 1.42 (95% CI − 1.65 to − 1.19). Ex vivo, the overall effect size was SMD − 0.56 (95% CI − 1.09 to − 0.03). Between-study heterogeneity (*I*^2^) was high for in vivo (72%, 95% CI 62 to 85%) and ex vivo studies (86%, 95% CI 78 to 98%). In vivo, in comparison to the overall effect size, hyperoxic vasoconstriction was less pronounced in the intestines and skin (*P* = 0.03) but enhanced in the cremaster muscle region (*P* < 0.001). Increased constriction was seen in vessels 15–25 μm in diameter. Hyperoxic constriction appeared to be directly proportional to oxygen concentration. For ex vivo studies, heterogeneity could not be explained with subgroup analysis.

**Conclusion:**

The effect of hyperoxia on vascular tone is substantially higher in vivo than ex vivo. The magnitude of the constriction is most pronounced in vessels ~ 15–25 μm in diameter and is proportional to the level of hyperoxia. Relatively increased constriction was seen in muscle vasculature, while reduced constriction was seen in the skin and intestines.

**Electronic supplementary material:**

The online version of this article (10.1186/s13054-018-2123-9) contains supplementary material, which is available to authorized users.

## Background

Superfluous oxygen supplementation in the acute, perioperative and intensive care setting frequently leads to arterial hyperoxia [1–3] and is associated not only with pulmonary side effects, but also with substantial acute hemodynamic changes. Hyperoxia can increase systemic vascular resistance due to (systemic) vasoconstriction and reduce cardiac output.

Although increased systemic vascular resistance indicates vasoconstriction, it does not inform in which organ the constriction occurs or at which location in the arterial tree. It is important for patient care to know whether constriction occurs equally or preferentially in organs. For instance, it may be argued that restriction of blood flow to resting skeletal muscle is less consequential than constriction in active cardiac muscle or organs like the liver and kidney. In animals, a hyperoxia-induced redistribution of blood flow to the kidney and splanchnic area has been observed, suggesting that vessels in these areas respond differently to hyperoxia [4–6].

Vascular diameter measurements in clinically relevant organs (e.g. visceral organs) often require surgical intervention. In humans, observations of hyperoxia-induced changes in vessel diameter in the microcirculation are therefore limited to superficial vascular beds such as the retina [7–9] and the sublingual vascular bed [10]. These organs are possibly not representative of hyperoxia-related vessel diameter changes in critical organs. Consequently, the effects of oxygen on vascular tone in deeper organs have been studied predominantly in animal models, using intravital microscopy (in vivo) and myography of isolated arteries (ex vivo). However, the results of these studies vary tremendously. Raising the oxygen tension to hyperoxic levels could lead to vasoconstriction [11–13], no effect [14–16] or even vasodilation [17, 18]. The causes of this heterogeneity are unclear, but might include differences between species, study methodology, vascular beds and/or the location of the hyperoxia sensor (e.g. the vessel wall or extravascular tissue). Identifying the source(s) of heterogeneity may contribute to a better understanding of the mechanism(s) involved in hyperoxic vasoconstriction [19].

With this systematic review and meta-analysis, we aim to provide an overview of the characteristics, quality and outcomes of all animal studies on the effect of hyperoxia on arteriolar tone, both in vivo and ex vivo. We also investigate the variation in magnitude of the hyperoxia effect among studies and explore sources of heterogeneity.

## Methods

This review is reported according to the preferred reporting items for systematic reviews and meta-analyses (PRISMA) guidelines [20]. The review methodology was specified in advance and documented using The Systematic Review Centre for Laboratory Animal Experimentation (SYRCLE) systematic review protocol for animal intervention studies ([21] and Additional file 1) and was registered on www.syrcle.nl on 22–02-2017. The protocol describes separate strategies for in vivo and ex vivo studies. However, due to considerable overlap and to enhance readability, we have combined the description of the review methodology where possible. The combined review question was: what is the effect of normobaric hyperoxia or hyperoxic superfusion on vascular tone of in vivo and ex vivo arteries and arterioles, in comparison to normoxia or normoxic superfusion?

### Amendments to the review protocol

To assess the risk of bias in studies, we planned to use the SYRCLE risk of bias tool. This tool has several items that relate to studies in which separate intervention and control groups are used. In such experiments, animal housing and animal selection may have a significant impact on outcomes. However, the studies included in this review (both in vivo and ex vivo) were all short-term pretest-posttest designs, performed in the same animal/vessels (e.g. no control groups). As a consequence, the SYRCLE risk of bias tool was not suitable for this review and was substituted by a customized quality checklist (see “Quality assessment” section). Due to the large variation in the size of the arteries investigated in the in vivo experiments, we added artery size post hoc as a possible source of heterogeneity (see “Data analysis”).

### Search strategy

We searched the databases Pubmed and Embase for articles published from inception up to 22 November 2017. The search strategy included various terms for vasoconstriction, vasodilation and hyperoxia. These categories were then combined with the AND operator and filtered for animal studies with the SYRCLE animal filter [22, 23]. We did not apply any language or date restrictions. Additional file 1 shows the search strategies used. We also checked the reference lists of included studies and relevant reviews for additional articles on hyperoxia that were not identified in the initial search.

### Selection of studies

Studies identified by the initial search were subjected to 3-phase screening, performed by two investigators (BS and AS). In the first phase, titles were screened to exclude studies not related to blood vessels or hyperoxia. In the second phase, titles and abstracts of articles were screened for eligibility based on the following predefined inclusion criteria: (1) controlled studies with repeated measures involving (2) adult healthy animals or isolated arteries or arterioles from adult healthy animals, (3) short-term normobaric, normocapnic hyperoxia in comparison with a normoxic control (see “Oxygenation definitions” for an explanation), (4) vessel diameter or tension measurements reported. In case of doubt, the full text of the publication was evaluated. Disagreements were resolved by discussion (BS and AS). In phase 3, full-text articles were assessed for final inclusion in more detail, based on the following exclusion criteria: (1) case reports, reviews and studies without an intervention, (2) disease models or models focusing on the vascular beds of the lung, brain or retina, the fetal vasculature or the ductus arteriosus, (3) studies without a focus on vasoreactivity, (4) vessels preconditioned by hypoxia, an intervention in the experimental design other than oxygen (e.g. addition of CO_2_, endothelium removal) or long term hyperoxia, (5) none of our predefined outcome measures were reported. In particular, the brain was excluded from analysis because it is known to possess extensive mechanisms for the regulation of its perfusion, therefore making it unsuitable for inclusion in meta-analysis of other more comparable vasculatures. Similarly, we excluded studies in non-healthy animals, to reduce anticipated heterogeneity because of altered regulation of perfusion during states of disease.

### Oxygenation definitions

For the in vivo studies, when the intervention was applied systemically, the measurements while the animal was breathing a gas with 20–21% O_2_ were considered as normoxic. Consequently, measurements during the inhalation of a gas with an oxygen content > 21% were recorded as hyperoxic. For in vivo studies where hyperoxia was established locally through superfusion, normoxia was defined as the state where tissue was superfused with a physiologic salt solution (PSS) equilibrated with 0–5% O_2_. With these concentrations, supply of oxygen to tissue by the PSS is limited as much as possible and occurs predominantly through the microcirculation. In these studies, hyperoxia was defined as superfusion with a PSS with an oxygen concentration of > 5%.

In ex vivo studies, partial pressure of oxygen (PO_2_) is directly comparable to partial pressure of oxygen in arterial blood (P_a_O_2_). Hence, the lower limit of normoxia was defined as PSS equilibrated with at least 7% O_2_, which yields a PO_2_ of ~ 55 mmHg. Because ex vivo oxygen exposures are often much higher than attainable in vivo (e.g. 100% O_2_ ex vivo gives a PO_2_ of ~ 760 mmHg, while inhalation of pure oxygen gives a P_a_O_2_ of approximately 350 mmHg), we used measurements at the lowest non-hypoxic oxygen exposure as the “normoxic” state and any exposures above as “hyperoxic”.

### Data extraction

From each article we extracted data on the species, strain, age, weight and sex of the animals used, type of anesthetic used, method and level of oxygen exposure, type of preparation/measurement setup, buffers used, descriptions of variability in preparations and oxygen dose-response relations. Reports on a change in vascular tone upon exposure to hyperoxia were extracted as “experiments” (e). Sometimes an experiment was not supported by data (e.g. data not shown). Experiments with data were extracted as “data sets” (k). These data sets include the number of animals/vessels used, mean and standard deviation or standard error of the vessel diameter (or equivalent, e.g. tension), or change from baseline data, during normoxic and hyperoxic exposures. Data not reported numerically were extracted from graphs with a digital ruler if possible. Because of incomplete outcome data in five studies, we contacted the corresponding author by email to request additional information. We received a response from one author, who was unable to supply the missing data. If a study reported multiple responses from similar vessels in separate groups (e.g. different unique control groups), then only the data from the group with the largest number of paired vessels were used in this meta-analysis. If there was uncertainty about the number of observations (e.g. the number of observations was reported as a range), the lowest value was used to calculate a conservative estimate of the standard error. All data were extracted by one author (BS) and then randomly checked by a second (AS).

### Quality assessment

The quality of studies was assessed using a tailor-made checklist containing ten items regarding blinding, preparation quality, verification of intervention efficacy, selective outcome reporting, description of animals and arteries, power analysis, ethical approval, conflicts of interest and peer review. For a list of the criteria used, see Additional file 2.

### Data analysis

All calculations were made using RStudio v1.1.383 and the “metafor” and “dosresmeta” package (Integrated Development for R. RStudio, Inc., Boston, USA). Graphs were made using GraphPad Prism 7.0 (GraphPad Software, Inc., La Jolla, USA) or RStudio. Effect sizes are presented as the standardized mean difference (SMD) for series of experiments involving paired comparisons as proposed by Gibbons [24]. All statistics are reported with their 95% confidence interval. An estimation of the variance of the SMD was done for a one group design with repeated measures [24]. Missing SD_difference_ values were calculated assuming correlation between measurements of 0.7 [25]. A sensitivity analysis using higher and lower correlation coefficients was conducted to test the robustness of our results. Effect estimates of the maximal hyperoxic exposure were pooled using a random effects model. Heterogeneity was assessed by the *I*^2^ statistic and is reported with its 95% confidence interval [26].

As a priori sources of heterogeneity we considered animal species, sex, vascular bed and method of hyperoxia exposure for in vivo studies. For ex vivo studies, we considered species, sex, tissue origin of the vessel, vessel type (systemic or resistance) and the presence of flow. We added baseline artery diameter post hoc as a possible source of heterogeneity for in vivo studies. The relation between effect size and artery diameter was explored through restricted cubic spline modeling, because it does not require any assumption on the type of relation between the two variables (*e.g* linear, sigmoidal etc.) [27]. The Akaike information criteria (AIC) was used to determine the optimal number of knots and their position [28]. For each subgroup a minimum of five data sets, from three unique studies, had to be present. Sources of heterogeneity were investigated using meta-regression, by first performing an overall test for interaction, and if the *P* value was <0.05, pairwise comparisons between subgroups were made to detect further subgroup interactions. To correct for multiple testing, *P* values were adjusted with the Holms-Bonferroni method.

The likelihood of publication bias was assessed using the trim and fill method [29]. Because SE-based precision estimates cause distortion of SMD funnel plots we used 1/√n as the precision estimate in the trim and fill analysis [30].

## Results

### Search and study selection

**Fig. 1:**
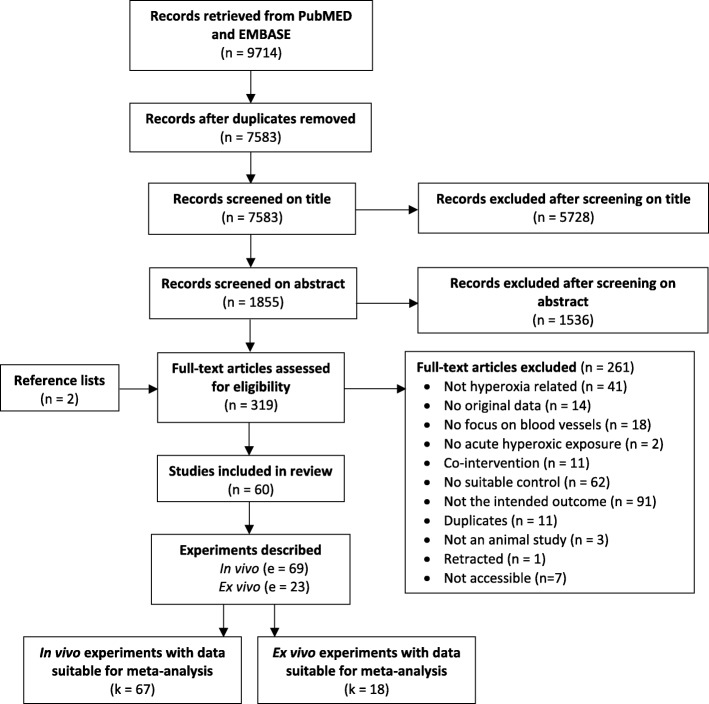
Flowchart describing the inclusion and exclusion of studies. n = number of studies, e = number of experiments described (either with or without data), k = number of data sets, which are the responses accompanied by data suitable for meta-analysis

A flow chart of the study selection process is shown in Fig. 1. After exclusion based on title and abstract, we identified 319 articles investigating the relation between oxygen and vascular tone: 261 studies were excluded based on predefined exclusion criteria. From the 60 included studies, 42 were performed in live animals and 18 studies used isolated arteries and arterioles. One study performed both eligible in vivo and ex vivo experiments [31]. Studies were performed between 1974 and 2017 and the majority (64%) was published between 1980 and 2000. Characteristics of the included studies are presented in Tables 1 (in vivo studies) and 2 (ex vivo studies).

**Table 1:** In vivo studies

1st Author, year of publication	Effect*	Species (strain)	Organ/tissue	Diameter (μm)	Vessels (n)	Oxygen**	
Duling, 1974 [38]	↓	Hamster (GS)	Cheek pouch muscle	15	14	0 /	21%
Gorczynski & Duling, 1978 # [61]	↓	Hamster	Cremaster muscle	13	3	0 /	10%
Davis et al., 1981 a [85]	↓	Hamster (GS)	Cheek pouch muscle	26	6	0 /	20%
Davis et al., 1981 b [85]	↓	Hamster (GS)	Cheek pouch muscle	14	6	0 /	20%
Davis et al., 1981 c [85]	↓	Hamster (GS)	Cheek pouch muscle	96	6	0 /	20%
Davis et al., 1981 d [85]	↓	Hamster (GS)	Cheek pouch muscle	51	6	0 /	20%
Lombard et al., 1981 a [86]	↓	Hamster	Cheek pouch muscle	19	10	0 /	10%
Lombard et al., 1981 b [86]	↓	Hamster	Cheek pouch muscle	9	7	0 /	10%
Sullivan & Johnson, 1981 a [36]	↓	Cat	Sartorius muscle	52	11	0 /	20%
Sullivan & Johnson, 1981 b [36]	↓	Cat	Sartorius muscle	35	16	0 /	20%
Sullivan & Johnson, 1981 c [36]	↓	Cat	Sartorius muscle	18	10	0 /	20%
Sullivan & Johnson, 1981 d [36]	↓	Cat	Sartorius muscle	13	6	0 /	20%
Sullivan & Johnson, 1981 e [36]	↓	Cat	Sartorius muscle	9	10	0 /	20%
Sullivan & Johnson, 1981 f [36]	↓	Cat	Sartorius muscle	8	12	0 /	20%
Jackson & Duling, 1983 [59]	↓	Hamster (GS)	Cheek pouch muscle	32	19	0 /	21%
Lombard & Stekiel, 1985 a [57]	↓	Rat (WS)	Mesoappendix	41	16	0 /	21%
Lombard & Stekiel, 1985 b [57]	↓	Rat (WS)	Mesoappendix	19	11	0 /	21%
Lombard & Stekiel, 1985 c [57]	↔	Rat (WS)	Mesoappendix	11	15	0 /	21%
Jackson, 1986 a [32]	↓	Hamster (GS)	Cheek pouch muscle	20	19	0 /	21%
Jackson, 1986 b [32]	↓	Hamster (GS)	Cremaster muscle	14	6	0 /	21%
Jackson, 1986 c [32]	↓	Rat (SD)	Cremaster muscle	19	8	0 /	21%
Jackson, 1987 [12]	↓	Hamster (GS)	Cheek pouch muscle	40	5	0 /	21%
Jackson, 1988 [62]	↓	Hamster (GS)	Cheek pouch muscle	31	22	0 /	21%
Lombard & Stekiel, 1988 a [66]	↓	Rat (WKY)	Mesoappendix	30	10	0 /	10%
Lombard & Stekiel, 1988 b [66]	↓	Rat (WKY)	Mesoappendix	18	15	0 /	10%
Lombard & Stekiel, 1988 c [66]	↓	Rat (WKY)	Mesoappendix	10	11	0 /	10%
Jackson, 1989 [63]	↓	Hamster (GS)	Cheek pouch muscle	28	19	0 /	21%
Bertuglia et al., 1991 a [43]	↓	Hamster (GS)	Dorsal skin	5	20	.21 /	1.0
Bertuglia et al., 1991 b [43]	↓	Hamster (GS)	Dorsal skin	7	12	.21 /	1.0
Bertuglia et al., 1991 c [43]	↑	Hamster (GS)	Dorsal skin	46	10	.21 /	1.0
Jackson, 1991 [67]	↓	Hamster (GS)	Cheek pouch muscle	12	11	0 /	21%
Taguchi et al., 1992 [87]	↓	Rabbit	Ear	?	6	.21 /	1.0
Jackson, 1993 a [64]	↓	Hamster (GS)	Cremaster muscle	16	22	0 /	21%
Jackson, 1993 b [64]	↓	Hamster (GS)	Cheek pouch muscle	20	15	0 /	21%
Rafi & Boegehold, 1993 a [33]	↓	Rat (SS/Jr)	Spinotrapezius	29	13	0 /	10%
Rafi & Boegehold, 1993 b [33]	↓	Rat (SS/Jr)	Spinotrapezius	17	17	0 /	10%
Rafi & Boegehold, 1993 c [33]	↓	Rat (SS/Jr)	Spinotrapezius	9	16	0 /	10%
Pries et al., 1995 [88]	↓	Rat (SD)	Spinotrapezius	35	18	0 /	20%
Dewhirst et al., 1996 [58]	↔	Rat (F344)	Dorsal skin	47	6	.21 /	1.0
Harder et al., 1996 [89]	↓	Rat (SD/WS)	Cremaster muscle	21	8	0 /	5%
Welsh et al., 1998 [60]	↓	Hamster (GS)	Cheek pouch muscle	52	17	0 /	21%
Lombard et al., 1999 a [90]	↓	Hamster (GS)	Cremaster muscle	19	19	0 /	21%
Lombard et al., 1999 b [90]	↓	Hamster (GS)	Retractor	22	11	0 /	21%
Lombard et al., 1999 c [90]	↓	Hamster (GS)	Cheek pouch muscle	21	10	0 /	21%
Frisbee & Lombard, 1999 [69]	↓	Rat (SD)	Cremaster muscle	20	12	0 /	21%
Frisbee & Lombard, 2000 [91]	↓	Rat (SD)	Cremaster muscle	17	12	0 /	21%
Frisbee et al., 2000 [92]	↓	Rat (WKY)	Cremaster muscle	18	6	0 /	21%
Sauls & Boegehold, 2000 [34]	↔	Rat (SD)	Ileum	63	11	5 /	21%
Komori et al., 2001 [68]	↓	Rabbit (Albino)	Ear	nr	11	.21 /	1.0
Kunert et al., 2001 a [73]	↓	Rat (SD)	Cremaster muscle	nr	16	0 /	21%
Kunert et al., 2001 b [72]	↓	Rat (WKY)	Cremaster muscle	nr	26	0 /	21%
Sauls & Boegehold, 2001 [35]	↔	Rat (SD)	Ileum	62	17	5 /	21%
Frisbee, 2002 [11]	↓	Rat (SD)	Cremaster muscle	22	12	0 /	21%
Frisbee & Lombard, 2002 [31]	↓	Rat	Cremaster muscle	102	12	0 /	21%
Tsai et al., 2003 a [45]	↓	Hamster (GS)	Dorsal skin	59	18	.21 /	1.0
Tsai et al., 2003 b [45]	↓	Hamster (GS)	Dorsal skin	25	15	.21 /	1.0
Tsai et al., 2003 c [45]	↓	Hamster (GS)	Dorsal skin	10	15	.21 /	1.0
Tsai et al., 2003 d [45]	↓	Hamster (GS)	Dorsal skin	6	15	.21 /	1.0
Drenjancevic et al., 2004 a [65]	↓	Rat (RGRR)	Cremaster muscle	19	8	5 /	21%
Drenjancevic et al., 2004 b [65]	↓	Rat (Dahl)	Cremaster muscle	22	8	5 /	21%
Cabrales et al., 2006 [44]	↓	Hamster (GS)	Dorsal skin	58	26	.21 /	1.0
Sakai et al., 2007 [37]	↓	Rat (WS)	Sciatic nerve	26	8	0 /	21%
Kunert et al., 2009 [93]	↓	Rat (SD)	Cremaster muscle	23	18	0 /	21%
Wang et al., 2009 [94]	↓	Rat (Dahl SS)	Cremaster muscle	16	9	0 /	21%
Ngo et al., 2010 [41]	↓	Mouse (C57)	Cremaster muscle	29	6	0 /	21%
Riemann et al., 2010 [42]	↓	Mouse (C57)	Cremaster muscle	31	7	0 /	95%
Messmer et al., 2012 [14]ǂ	↔	Hamster (GS)	Dorsal skin	nr	?	.21 /	1.0
Ngo et al., 2013 [40]	↓	Mouse (C57)	Cremaster muscle	33	28	0 /	21%
Milstein et al., 2016 [39]	↓	Rabbit (NZW)	Sublingual	4	40	.21 /	1.0

Studies are sorted based on year of publication

*Abbreviations: NZW* New Zealand White, *SD* Sprague Dawley, *WS* Wistar, *LY* Landrace Yorkshire, *M* muscle chamber, *W* wire myograph, *P* pressure myograph, *nr* not reported

*Effect as reported by the original paper, arrows indicate the direction of the change in diameter

**A value suffixed with a “%” symbol indicates the percentage of oxygen used to oxygenate the physiological salt solution; other values indicate the fraction of inspired oxygen

#Not included in the meta-analysis due to a small number of observations or because no data was shown (ǂ)

**Table 2:** Ex vivo studies

1st Author	Effect*	Species (strain)	Artery	Type	Tone	Model	Diameter (μm)	Vessels (n)	Oxygen**	
Chang & Detar, 1980 [54]	↓	Rabbit (NZW)	Aorta	Conduit	Norepinephrine	M	4000–5000	10	100 /	300
Pittman & Graham, 1986 a [55]ǂ	↓	Rabbit (NZW)	Aorta	Conduit	Norepinephrine	M	nr	?	±100 /	280
Pittman & Graham, 1986 b [55]ǂ	↓	Rabbit (NZW)	Aorta	Conduit	KCl	M	nr	?	±100 /	280
Vallet et al., 1994 [56]	↓	Rat (SD)	Aorta	Conduit	Phenylephrine	M	2200–2800	6	20 /	95%
Day et al., 1992 [51]	↔	Rabbit (NZW)	Aorta	Conduit	None	W	nr	6	160 /	495
Kwan et al., 1989 [52]	↑	Sheep	Coronary	Conduit	None	M	600–2500	8	12 /	95%
Ngai et al., 1990 [17]	↑	Pig	Coronary	Conduit	None	M	nr	10	20 /	95%
Rubanyi & Paul, 1984 b [15]	↑	Pig	Coronary	Conduit	Histamine	M	nr	6	40 /	95%
Rubanyi & Paul, 1985 [18]	↑	Pig	Coronary	Conduit	Spontaneous/KCl	M	1500–2000	10	12 /	95%
Hedegaard et al., 2011 [70]	↓	Pig (LY)	Coronary	Conduit	PGF2a	W	nr	9	10 /	95%
Kalsner, 1976 [46]	↓	Cow	Coronary	Conduit	Spontaneous	M	nr	14	112 /	515
Pasgaard et al., 2007 b [53]	↓	Pig	Coronary	Conduit	U46199	W	nr	14	120 /	617
Pasgaard et al., 2007 c [53]	↓	Pig	Coronary	Conduit	5-HT	W	nr	7	120 /	617
Pasgaard et al., 2007 a [53]	↔	Pig	Coronary	Conduit	None	W	nr	?	120 /	617
Rubanyi & Paul, 1984 a [15]ǂ	↔	Cow	Coronary	Conduit	KCl	M	nr	?	40 /	95%
Frisbee & Lombard, 2002 [31]#	↓	Rat	Cremaster	Resistance	Spontaneous	P	140	12	10 /	21%
Messina et al., 1994 [13]	↓	Rat (WS)	Cremaster	Resistance	Spontaneous	P	163	27	21 /	95%
Smit et al., 2017 b [16]	↔	Mouse (C57)	Femoral	Conduit	Norepinephrine	P	293	16	78 /	375
Fredricks et al., 1994 [47]	↓	Rat (SD)	Gracilis	Resistance	Spontaneous	P	100–300	7	87 /	148
Frisbee et al., 2001 [48]	↓	Rat (SD)	Gracilis	Resistance	Spontaneous	P	185	15	15 /	95%
Frisbee et al., 2002 [49]	↓	Rat (SD)	Gracilis	Resistance	Spontaneous	P	194	76	10 /	21%
Liu et al., 1997 [50]	↓	Rat (SD)	Gracilis	Resistance	Spontaneous	P	nr	6	10 /	21%
Smit et al., 2017 a [16]	↔	Mouse (C57)	Gracilis	Resistance	Spontaneous	P	129	22	78 /	375

Studies are sorted based on artery, effect and first author

*Abbreviations: NZW* New Zealand White, *SD* Sprague Dawley, *WS* Wistar, *LY* Landrace Yorkshire, *M* muscle chamber, *W* wire myograph, *P* pressure myograph, *nr* not reported

*Effect as reported by the original paper; arrows indicate the direction of the effective change in diameter

**A value suffixed with a “%” symbol indicates the percentage of oxygen used to oxygenate the physiological salt solution, other values indicate the oxygen tension in mmHg

ǂNot included in the meta-analysis because no data was shown or values could not be recalculated (#). For diameters, values are presented as range or average diameter

### Study characteristics

#### In vivo studies

The 42 in vivo studies were carried out in rats (*n* = 19), hamsters (*n* = 17), mice (*n* = 3), rabbits (n = 3) and cats (*n* = 1). In one study both rats and hamsters were used [32]. Most studies exclusively used male animals (*n* = 27). In one study both male and female animals were used, in one only females were used and in the remaining studies the sex of animals was not reported. Rats were between 5 and 18 weeks of age and weighed 75–413 g (range of study averages). Ages were not reported for other animals. Hamsters weighed 55–200 g, mice 24–35 g, rabbits 3–4 kg and cats 0.9–1.5 kg. In most studies the animals were anesthetized with pentobarbital (*n* = 28, used predominantly in rats and hamsters). Other anesthetics were thiopental [33–35], chloralose [36, 37], urethane [37, 38], ketamine [39] or a mixture of droperidol, midazolam and fentanyl [40–42]. In some studies skinfold windows were applied so that the microcirculation could be observed without anesthesia [14, 43–45].

Each study reported 1–6 experiments in which blood vessel diameter was observed while hyperoxia was induced. Combined, a total of 69 experiments (e) were described in these studies, which included measurements on arterioles with a median diameter of 20 μm (interquartile range 14–34 μm, 1st to 6th order arterioles) in the cremaster (*e* = 20), cheek pouch (*e* = 16), dorsal skin (*e* = 10), sartorius muscle (*e* = 6), mesoappendix (*e* = 6), spinotrapezius muscle (*e* = 4), ear (*e* = 2), ileum (*e* = 2), retractor muscle (*e* = 1), sciatic nerve (*e* = 1) and the sublingual microcirculation (*e* = 1). The area of interest was superfused with a physiologic salt solution, equilibrated with 0% or 5% O_2_ (normoxia, *e* = 52 and *e* = 4, respectively) and subsequently exposed to 10% (hyperoxia, *e* = 9), 21% (*e* = 46) or 95% O_2_ (*e* = 1). In 13 experiments, blood vessels were observed through a surgically implanted window (*e* = 7, in the cheek pouch or the ear) or non-invasively by a hand-held device (*e* = 1), while hyperoxia was induced systemically by increasing the fraction of oxygen inspired by the animal (*e* = 13).

#### Ex vivo studies

In the 18 ex vivo studies, isolated arteries from rats (*n* = 7), pigs (*n* = 4), rabbits (*n* = 3), cows (*n* = 2), mice (*n* = 1) and sheep (n = 1) were used. In 10 studies male animals were used and in one study animals of either sex were used [15]: in the remaining 7 studies animal sex was not reported. Rats were 6–15 weeks old and weighed 200–500 g. Pigs were about 4 months old and weighed 70–100 kg. Mice were 10–12 weeks old and weighed 25 g on average. The age and weight of cows and sheep were not reported.

In these studies, 23 experiments were described. The maximal diameters of the vessels ranged between 100 and 5000 μm and they were exposed to hyperoxia in muscle chambers (*e* = 10), wire myographs (*e* = 5) and pressure myographs (*e* = 8). Both conduit (thoracic aorta, femoral artery, coronary artery) and resistance arteries (gracilis arteriole, cremaster arteriole) were investigated. All studies used physiological salt solutions with comparable contents. After isolation, the blood vessels in some studies spontaneously developed tone [13, 16, 18, 31, 46–50]. Isolated vessels that did not develop tone were left unconstricted [17, 51–53] or were constricted to ~ 40–50% of their baseline diameter with norepinephrine [16, 54, 55], phenylephrine [56], histamine [15], potassium chloride [15, 18, 55], thromboxane (U46199) [53], serotonin (5-HT) [53] or prostaglandin F2α [53]. Vessels were exposed to normoxia by gassing the surrounding buffer with 10–21% oxygen. In one study, 40% oxygen was used in the control group [15]. Hyperoxia was induced by changing the gas supply to 21% or 95% O_2_. In 11 experiments, the authors measured the PO_2_ of the solutions, which ranged from 78 to 160 mmHg for normoxia and 280–617 mmHg for hyperoxia.

#### Quality assessment

**Fig. 2:**
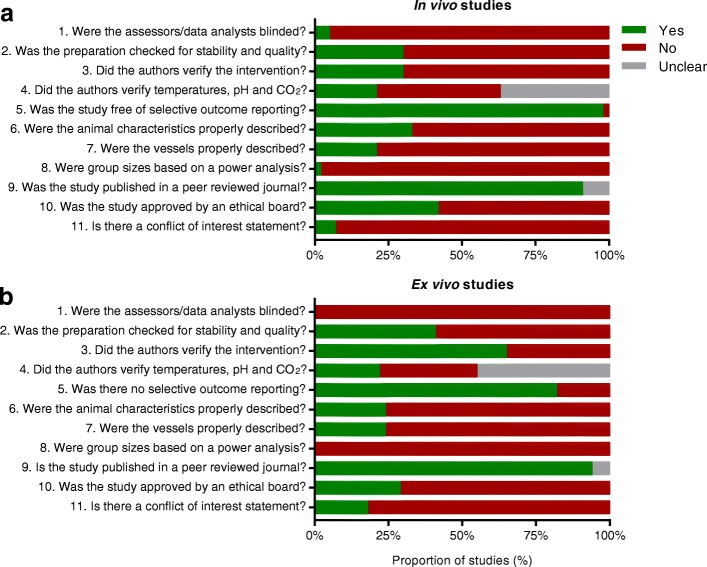
Quality assessment scores for in vivo studies (**a**) and ex vivo studies (**b**)

See Fig. 2 for an overview of the quality assessment. Overall, the reporting of key quality indicators was poor in both in vivo and ex vivo studies. Only 2 out of 60 studies (3%) reported blinding of any phase of the experiment; these two cases were in vivo studies where the outcome assessor was blind to the experimental groups. Quality checks of the preparation were not described in 66% of the studies and 62% of the studies did not report whether and how the intervention was verified (e.g. by measuring PO_2_ levels). Similarly, the reporting of other preparation conditions (temperature, CO_2_ and pH) was insufficient in 79% and 78% of the studies, respectively. Adequate descriptions of relevant details about the animals (e.g. sex, number of animals used), or vessels (diameters, selection) were lacking in 70% and 78% of the studies, respectively. Only one study performed a power analysis. The majority of studies (66%) were published in peer-reviewed journals, however, for 4 studies it was unclear whether these were peer-reviewed. Only 6 studies (10%) provided a conflict of interest statement and only 24 studies (40%) mentioned approval by an ethical board.

### Descriptive synthesis of outcomes

#### In vivo effect of hyperoxia on vascular tone

According to the effects reported by the original authors (Table 1), raising the P_(a)_O_2_ to hyperoxic levels resulted in vasoconstriction in 91% of the studies. In 7% there was no effect on arteriolar diameter [35, 57, 58] and in one study dilation of second order arterioles was observed [43]. The median diameter change was − 20% with a range of − 64% to + 6%.

#### Ex vivo effect of hyperoxia on vascular tone

Including the experiments without data, hyperoxic vasoconstriction was seen in 59% of the reports. In 23% there was no change in arteriolar diameter [15, 16, 51, 53] and in 18% dilation was observed [15, 17, 18, 52]. In the studies that reported diameters [13, 16, 47–50], the median change was − 14% with a range of − 24% to − 1%.

#### Experimental difficulties

Some articles explicitly mentioned difficulties encountered during the experiments and variation in responses between separate vessels. In vivo, a vasomotor response to changes in oxygen exposure was not observed in 12% [38], 25% [59] and 40% [42] of the animals. The authors did not provide an explanation for non-responsiveness in these animals. In two studies, 26–50% of the vessels failed to return to their stable baseline diameter after the first exposure to hyperoxia [41, 60].

Ex vivo, one study described that only 32% of the isolated arteries responded to oxygen [59]. In addition, in reactive vessels, the constriction to oxygen disappeared after 2–6 oxygen exposure cycles. The author concluded that the lack of oxygen reactivity was not an indication of vessel damage, because both responding and non-responding vessels developed spontaneous tone and had similar responses to norepinephrine. In porcine coronary arteries, repeated exposures to hyperoxia also decreased the magnitude of hyperoxic vasoconstriction.

#### Dose-response studies

Dose-response relationships were examined in 16 in vivo studies: 10 of these used three oxygen concentrations [39, 41, 57, 61–67] (including reference level), in four studies four levels of oxygen were used [59, 68, 69] and in two studies five different oxygen concentrations were used [11, 31]. In the majority of these studies, 20–21% oxygen in the superfusion buffer was the maximal exposure, while one study also used 95% oxygen [59]. Intermediate oxygen concentrations were 5%, 7% or 10% oxygen. In studies with more than three different oxygen doses, the relationship between diameter changes and oxygen concentration was approximated as sigmoidal [11], exponential [59, 68] or linear [31, 69]. In general, arteriolar diameter decreased as the oxygen concentration increased, without any apparent threshold dose. Only in one study, in which the relationship seemed sigmoidal [11], no constriction occured until the oxygen concentration in the superfusion buffer was increased above 5%.

Of the ex vivo studies, eight studies used a total of three [18, 31, 47, 49], four [16, 50, 70] or five [55] oxygen concentrations (including reference level). The range of oxygen exposure assessed varied between 10 and 21% to 10–95%. In all studies except for two, a decrease in diameter was seen as the oxygen concentration increased. The dose-response relationships in studies with more than three levels of exposure were described as linear in the normoxic-hyperoxic range [16, 55, 70, 71]. In one study there was no discernable difference between 40% or 95% O_2_ [18] and in another there was no effect at any oxygen concentration (10–95% O_2_) [16].

### Meta-analysis

#### In vivo studies

**Fig. 3:**
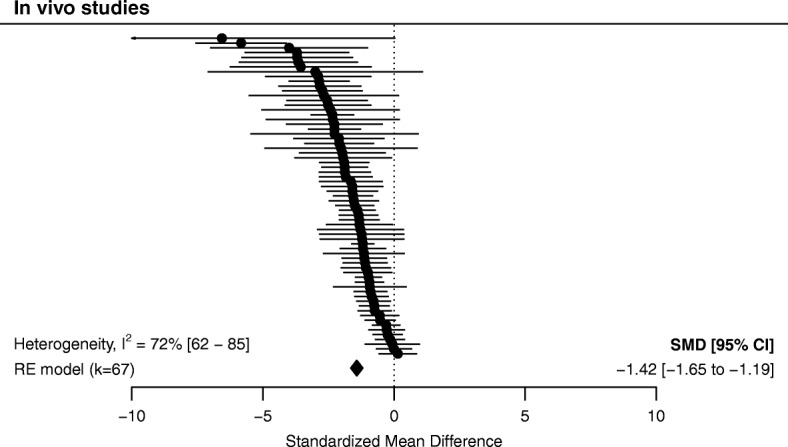
In vivo studies on the effect of hyperoxia on arteriolar diameter, sorted by effect size

Out of the 69 experiments, 67 (k = 67) were included in the meta-analysis of in vivo studies. Two could not be included because in one study only three vessels were observed [61] and in another the data were not shown [14]. Overall, there was a significant vasoconstrictor effect of hyperoxia on arteries in vivo (SMD − 1.42 (− 1.65 to − 1.19), Fig. 3); heterogeneity between studies was high (*I*^2^ 72% [62–85]).

#### Ex vivo studies

**Fig. 4:**
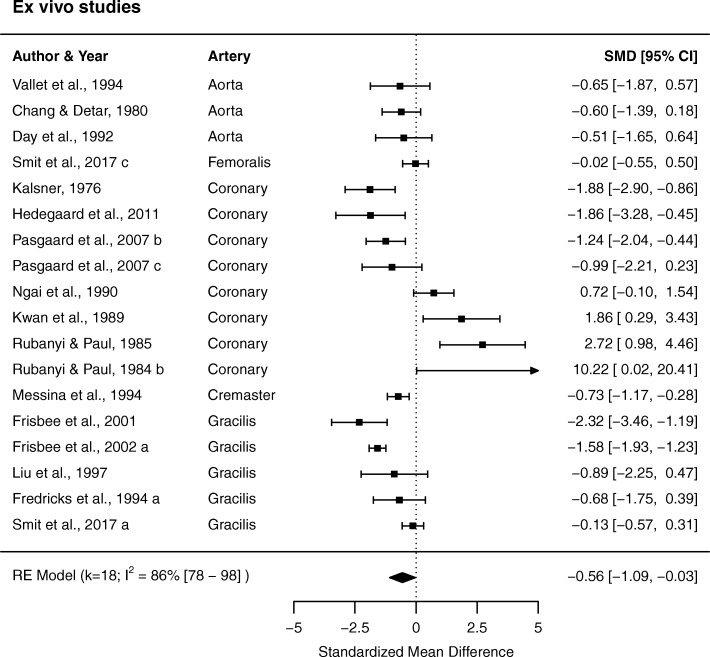
Data sets from ex vivo studies on vascular diameter or tone. The plot is sorted by artery type and effect size. SMD, standardized mean difference

Out of the 23 experiments, 18 (k = 18) were included in the meta-analysis of ex vivo studies. Four experiments were mentioned in the text but were not accompanied by data [15, 53, 55] and one paper reported normalized data for one experiment that could not be appropriately recalculated [31]. Overall, there was a borderline significant vasoconstrictor effect of hyperoxia on arteries ex vivo (SMD − 0.56 (− 1.09 to − 0.03), *P* = 0.04, Fig. 4). Between-study heterogeneity was high (*I*^2^ 88% [80–87]).

### Exploring sources of heterogeneity

#### In vivo studies

**Fig. 5:**
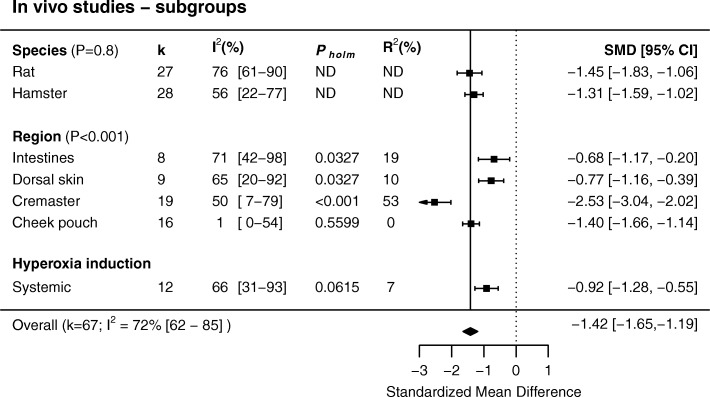
Subgroup analyses of in vivo studies. Hyperoxia induced more constriction in the cremaster vasculature. Hyperoxic vasoconstriction was reduced in the dorsal skin and intestines (mesoappendix and ileum). *P*_*holm*_ values are corrected for multiple testing with the Holm-Bonferroni method. ND, not determined; k, data sets

**Fig. 6:**
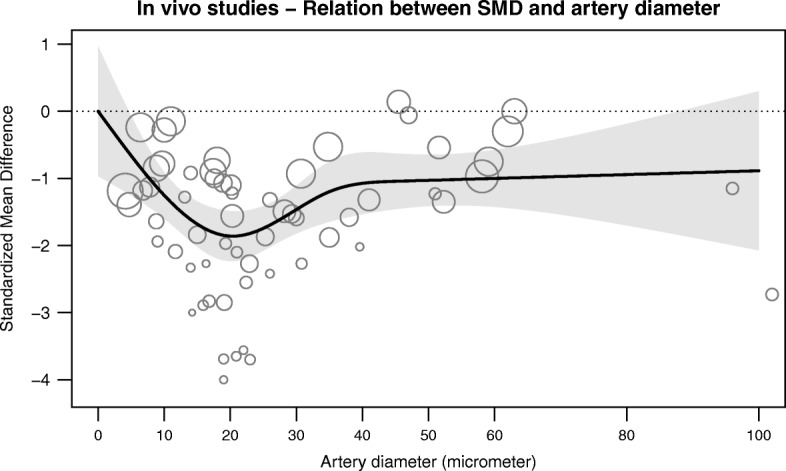
Relationship between artery diameter and effect size. Increased constriction was seen in the range of 15–25 μm. The relationship between the standardized mean difference (SMD) and artery diameter explained 16% of the observed heterogeneity in the overall effect

Subgroup analyses were only conducted in groups with five or more experiments from at least three unique studies. For the in vivo studies, subgroup analyses were conducted for species (rat and hamster, Fig. 5), vascular region (dorsal skin, intestines, cremaster muscle, cheek pouch, Fig. 5), method of hyperoxia induction (systemic by increasing the percentage of oxygen in the inspired air, Fig. 5) and vessel diameter (Fig. 6). Sex could not be examined because the studies mostly used male animals or did not specifically report animal sex. Species did not modify the overall effect (*P* = 0.83). Vascular region interacted with the overall effect (*P* < 0.001). *P* values for further pairwise comparisons were adjusted with the Holms-Bonferroni method. Moderators that increased the effect size of hyperoxic constriction were the cremaster as a vascular region (*P*_*holm*_ < 0.001). This subgroup explained 53% of the heterogeneity (*R* [2]) in the overall effect. A smaller response was seen in studies in which the dorsal skin (*P*_*holm*_ = 0.033, *R*^2^ = 10%) or the intestines was investigated (mesoappendix and ileum, *P*_*holm*_ = 0.033, *R*^2^ = 19%). The effect size was not different in studies that induced hyperoxia systemically (*P*_*holm*_ = 0.061, *R*^2^ = 7%). In a weighted four-knot restricted cubic spline meta-regression, artery diameter accounted for 16% of the observed heterogeneity (*P* = 0.008, Fig. 6).

#### Ex vivo studies

**Fig. 7:**
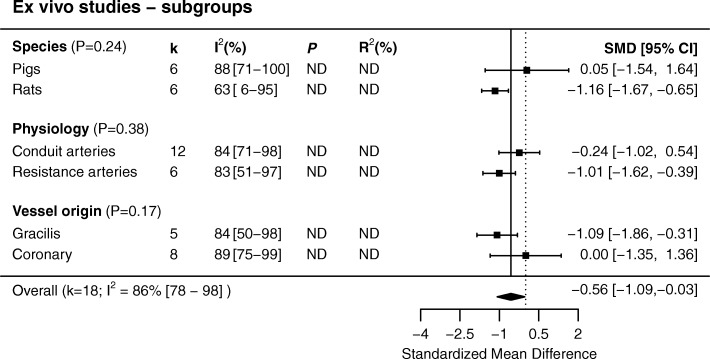
Subgroup analyses for ex vivo studies. None of the investigated subgroups explained a significant proportion of the heterogeneity. K, data sets; SMD, standardized mean difference

In the ex vivo studies, only two species were used in a sufficient number of studies to be considered as subgroups (pigs and rats). Other potential sources of heterogeneity investigated were type of artery (conduit or resistance arteries) and vessel origin (gracilis and coronary arteries). Coincidentally, all conduit arteries were examined in tension myographs, while resistance arteries were mounted in pressure myographs. Hence, results of an analysis based on the method used to mount the isolated arteries would be the same as now presented based on the type of artery. There were not sufficient studies reporting sex or using flow to analyze as subgroups. Of the investigated subgroups, none explained a significant proportion of the heterogeneity (Fig. 7).

### Publication bias and sensitivity analysis

The funnel plot for the in vivo studies shows an indication of publication bias, favoring studies that reported constriction of arterioles upon exposure to hyperoxia (Additional file 3). Balancing the results with the trim and fill method reduced the overall effect size (*P* = 0.03) from − 1.42 (− 1.65 to − 1.19) to − 1.03 (− 1.30 to − 0.76). The funnel plot for the ex vivo studies did not reveal evidence of publication bias (Additional file 3).

For all analyses shown, we used a conservative correlation coefficient of 0.7 to impute missing standard deviations of paired data. Missing standard deviations had to be calculated for 21/67 in vivo data sets and 7/18 ex vivo data sets. Using a higher or lower correlation coefficient did not affect the results for the in vivo analysis (Additional file 4). For the ex vivo analysis, the differences were marginal, but when a low correlation coefficient of 0.5 was used, the confidence interval of the overall effect crossed zero.

## Discussion

In this systematic review and meta-analysis, we found that normobaric hyperoxia or hyperoxic superfusion decreases the diameter of in vivo arteries and arterioles, in comparison to normoxia or normoxic superfusion, indicating an increase in vascular tone. For ex vivo studies, our meta-analysis shows a smaller effect of hyperoxia decrease in isolated vessel tone. Both vasodilation, vasoconstriction and neutral effects were observed, for which the explanation is unclear. In contrast, in the intact animal hyperoxia almost exclusively causes vasoconstriction (median − 20% reduction in diameter), which was most pronounced in the cremaster vasculature but less in the intestinal and skin vasculature. In a post-hoc analysis, the magnitude of hyperoxic vasoconstriction showed a U-shaped curve and was highest in vessels of 15–25 μm in diameter. There seems to be no oxygen threshold for the constriction to occur.

There was a large difference in effect size between the in vivo and ex vivo studies. The existence of a branching order-specific effect, i.e. confinement of hyperoxic vasoconstriction to smaller arterioles, could be one explanation for this difference. We found that oxygen-induced constriction was the largest in arteries of 15–25 μm in diameter and less in arteries with diameters above and below this range. The arteries used in the ex vivo studies were all considerably larger than the vessels examined in vivo, ranging between 100 μm and 5000 μm ex vivo, versus 4–100 μm in vivo. The difference in size between the investigated arteries is the result of a practical limitation, considering that it is very difficult to isolate and mount small arteries without damaging the vessel ex vivo. The reduced effect of hyperoxia on arteries < 20 μm may be explained by the decreasing presence of vascular smooth muscle and thus the ability to constrict. Another explanation is that the mechanism responsible for hyperoxic vasoconstriction is primarily located in extravascular tissue, rather than in the vessel wall. For example, the CYP450 omega hydroxylases, which can produce the vasoconstrictor 20-hydroxyeicosatetraenoic acid (20-HETE) from arachidonic acid, is found in cremaster arterioles, but is also highly expressed in the surrounding muscle tissue [72, 73]. Hence, for substantial (measurable) hyperoxic vasoconstriction, extravascular tissue may be required.

We found regional differences in the magnitude of hyperoxic constriction between the intestine, skin and cremaster muscle. The differences are likely related to intrinsic differences in blood vessels between vascular beds, or presence of multiple mechanisms that regulate vascular tone. For instance, 20-HETE constricts arteries in the cremaster and gracilis muscle but dilates mesenteric arteries [74]. Inhibition of nitric oxide in intestinal arteries only temporarily reduces vessel diameter [35], which suggests that in these vessels the impediment of one vasoactive system is quickly compensated by another. The same might be true for the pathway affected by hyperoxia. The reduced effect in skin could be related to the already low basal perfusion of skin at room temperature, so that any further reductions in flow and artery diameter are prevented by other mechanisms, such as temperature control [75]. In humans, hyperoxia has no effect on skin flow, unless first pharmacologically raised or measured in existing high-flow areas. Because control of body temperature is a major aspect of skin perfusion, the pathway for temperature control could take precedence over the oxygen sensor. The exact mechanism behind hyperoxic vasoconstriction has not yet been determined. Potential pathways include a reduction in nitric oxide bioavailability due to increased reactive oxygen species production, decreased production of prostaglandins, increased production of 20-HETE and interaction with calcium and potassium channels. The varying results may not necessarily be conflicting but could be simply due to differences in vascular beds and locations in the arterial tree.

This meta-analysis shows that hyperoxia in vivo causes a relatively increased level of vasoconstriction in muscle (i.e. cremaster and cheek pouch). Although perfusion of skeletal muscle is arguably not a priority in the critically ill, a restriction of blood flow in cardiac muscle may be detrimental. In one trial, hyperoxia led to increased infarct size in patients with myocardial infarction [76]; this may have been the result of decreased perfusion, considering that in pigs, hyperoxic coronary vasoconstriction leads to increased tissue hypoxia in areas with acute coronary stenosis [77]. The reduced constriction observed in the intestinal region could be of clinical importance. Organs with relatively reduced hyperoxic constriction will receive a larger portion of the cardiac output. This has been observed in hemodiluted dogs, pigs with peritonitis and rats with hemorrhagic shock, where hyperoxia caused a redistribution of the cardiac output towards vital organs such as the kidney, liver and intestines [4, 5, 78]. In rats, hyperoxia was shown to reduce bowel injury after ischemia and reperfusion of the mesentery [79]. Because of the relative increase in perfusion, hyperoxia may thus be beneficial for patients with splanchnic hypoperfusion.

Vessel diameter has a dominant influence on blood flow. In vivo, the median hyperoxia-induced decrease in diameter was 20%, which, using Poiseuille’s law, leads to a reduction of approximately 60% in flow. Following the dose-response studies, the constrictor effect of oxygen occurs at any level above normoxia, meaning that any level of hyperoxia will have consequences for organ blood flow if vasoconstriction occurs. However, the translatability of these effects to clinical practice is difficult. The effect of oxygen on artery diameter depends on oxygen tension, which was rarely measured in the included studies. The oxygen percentages used in superfusion buffers cannot be directly converted to a fraction of inspired oxygen. Second, in the majority of the in vivo studies, both the arteries and tissue were exposed to the hyperoxic oxygen tensions, meaning that the oxygen delivery was no longer solely facilitated by blood flow. Any tissue-derived signals generated by the reduction in blood flow may therefore have been missed, confounding the results.

Human hyperoxic coronary vasoconstriction is a well-documented phenomenon and has been observed directly by means of angiography [80, 81] and indirectly via increases in coronary vascular resistance measurements [80, 82–84]. Surprisingly however, results of ex vivo studies of the isolated coronary circulation are less univocal. Of the eight experiments with coronary vessels, vasoconstriction to increased oxygen tensions was seen in four, while vasodilation was observed in the other four. Differences could again be related to vessel size. In the studies in which coronary constriction was observed, arteries were mounted in wire myographs, which are suitable for vessels measuring > 100 μm. Unfortunately, these studies did not report vessel diameter. However, the studies in which dilation was seen used muscle chambers, which are generally used for large arteries. Two of these studies reported diameter, which ranged between 0.6 and 5 mm. Another important consideration is that constriction was only seen with certain types of preconstriction. This was especially apparent in one study where coronary preparations were used without preconstriction or were constricted with serotonin (5HT) or thromboxane (U46619) analogues [53]. Hyperoxia had no effect on vessels without preconstriction, while a considerable hyperoxic constriction was seen in the precontracted vessels. Taken together; vessel preparation may be particularly important in ex vivo experiments and the use of isolated vessels may not be appropriate to study the complex vascular responses to hyperoxia.

### Limitations of this review and included studies

There are several limitations to this meta-analysis. There was considerable heterogeneity between the in vivo studies, which could only in part be explained by differences in the region of interest and the size of the vessels investigated. For the ex vivo studies we did not find an explanation for the heterogeneity, suggesting the importance of a yet unidentified methodological, systemic or metabolic factor. Second, the funnel plots indicated publication bias for in vivo studies, favoring studies finding vasoconstriction in response to hyperoxia. However, correction with the trim and fill method did not significantly alter the conclusions. Third, due to the relatively small number of studies, some collinearity between the investigated subgroups may be present. Fourth, for some studies we had to make an assumption on the correlation coefficient to impute missing standard deviations. Our conservative estimate may have underestimated the effect, although our sensitivity analysis shows that using a higher correlation coefficient did not significantly alter the results. Fifth, all studies were performed with healthy animals; studies in disease models may give different results. Similarly, the use of live animals required the application of anesthetics in the majority of studies. Most of these drugs have at least some vasodilatory effects, which may have influenced the results. However, because of the pre-post design of the included studies, the effect of the anesthetic is anticipated to be the same under each oxygen condition. Sixth, we excluded studies on the lung, retinal and brain vasculature.

Finally, as reflected by our quality assessment of the included studies, the overall quality of the included studies was poor, which seriously hampers drawing reliable conclusions from the included animal studies. Baseline characteristics of animals were insufficiently described. Similarly, only 14 of the 60 studies properly described artery characteristics, such as vessel diameter and selection of vessels in the arterial tree. Reporting these data is important to increase reproducibility between studies. Similarly, measurement of PO_2_, pH, PCO_2_ and temperature was not (sufficiently) performed in the majority of studies. These measurements are crucial because of the dose response relationship between PO_2_ and hyperoxic vasoconstriction, and the effect of pH, PCO_2_ and temperature on vascular tone. Without measuring PO_2_ the true intervention applied to the vasculature is unknown, which will contribute to heterogeneity. The lack of blinding in most studies may have led to an overestimation of the true effect. Another flaw in most studies is the absence of power calculations. Especially for studies aimed at discovering mechanisms responsible for oxygen-related changes in vessel diameter, a minimum predefined number of vessels is necessary to be able to draw objective statistical conclusions. The majority of the included studies were conducted before the institution of more rigorous legislation on the use of animals in scientific procedures known today. The overall poor quality of studies is therefore likely related to the absence of an ethical board reviewing the studies prior to their execution. Similarly, the same papers were published before editors put certain requirements on the reporting of study data. We have noted that the more recent papers were of considerably higher quality, which shows that the change in procedures concerning animal research, which are now becoming very similar to human trials in terms of ethical board review and data transparency, is for the better.

The majority of the in vivo studies were performed on thin, externalizable skeletal muscle. Studies in other critical visceral organs (e.g. liver, kidney) are currently lacking. We propose that future, high-quality studies focus on visceral organs such as the kidney and liver. These organs are highly relevant for patient care and the results will contribute to the rationale behind selective induction or avoidance of hyperoxia in certain types of patients.

## Conclusions

In this meta-analysis of in vivo and ex vivo studies investigating the effect of hyperoxia on arteriolar diameter, we found a substantially larger effect size in vivo than ex vivo. The degree of the constriction is most pronounced in vessels of approximately 15–25 μm in diameter and is proportional to the level of hyperoxia. The vasculature of the intestines and skin seem less sensitive to hyperoxic vasoconstriction than skeletal muscle. Ex vivo, the response of the coronary vasculature to hyperoxia is highly variable.

## Additional files

Additional file 1: Search strategy. The search strategies used to search Pubmed and Embase for eligible studies. (PDF 201 kb)

Additional file 2: Quality checklist. Modified quality checklist used to assess the quality of the included studies. (PDF 14 kb)

Additional file 3: Funnel plots. Results of the publication bias assessment in the form of funnel plots. (PDF 344 kb)

Additional file 4: Sensitivity analysis. Results of the sensitivity analysis for the correlation coefficients. (PDF 232 kb)

## Abbreviations

20 HETE: 20-Hydroxyeicosatetraenoic acid
AIC: Akaike information criteria
CI: Confidence interval
e: Experiments
EDHF: Endothelium dependent hyperpolarizing factors
EDRF: Endothelium derived relaxing factors
FIO2: Fraction of inspired oxygen
k: Datasets
PaO2: Partial pressure of oxygen in arterial blood
PO2: Partial pressure of oxygen
SD: Standard deviation
SMD: Standardized mean difference
SYRCLE: The Systematic Review Centre for Laboratory Animal Experimentation
