# Supplementary material for: Effects of hyperoxia on vascular tone in animal models: systematic review and meta-analysis
Source: Crit Care. 2018 Aug 4;22:189. doi: 10.1186/s13054-018-2123-9 (PMC6091089; doi:10.1186/s13054-018-2123-9)
Supplement: Supplementary file 1 — Search strategy. The search strategies used to search Pubmed and Embase for eligible studies. (PDF 201 kb) [file 13054_2018_2123_MOESM1_ESM.pdf]

Supplemental File 1 – Search strategy

PubMed query

Vasoconstriction/vasodilatation

“vasoconstriction” [MeSH Terms] OR “vasomotor system” [MeSH Terms] OR “vasodilation” [MeSH Terms] OR "Arteries/drug effects"[Mesh] OR “vasoconstriction” [tiab] OR “vasoconstrictions” [tiab] OR “vasoconstrictor” [tiab] OR “vasoconstrictors” [tiab] OR “vasopressor” [tiab] OR “vasoactive agonist” [tiab] OR “vasoactive agonists” [tiab] OR “vasopressors” [tiab] OR “vasomotor system” [tiab] OR “artery constriction” [tiab] OR “vessel constriction” [tiab] OR “vasoconstrictive” [tiab] OR “vasoconstricting” [tiab] OR “vasoconstricted” [tiab] OR “vasodilation” [tiab] OR “vasodilatation” [tiab] OR “vasodilatating” [tiab] OR “vasodilating” [tiab] OR “vasodilative” [tiab] OR “vasodilatative” [tiab] OR “artery dilation” [tiab] OR “vessel dilation” [tiab] OR “artery dilatation” [tiab] OR “vessel dilatation” [tiab] OR “vasodilator” [tiab] OR “vasodilators” [tiab] OR “vasorelaxation” [tiab] OR “Vascular Endothelium Dependent Relaxation” [tiab] OR “Endothelium Dependent Relaxation” [tiab] OR “Vascular Endothelium-Dependent Relaxation” [tiab] OR “Endothelium-Dependent-Relaxation” [tiab] OR “vasodilated” [tiab] OR “vasoactive agent” [tiab] OR “vasoactive drug” [tiab] OR “vasoactive drugs” [tiab] OR “dilation” [tiab] OR “dilatation” [tiab] OR “contraction” [tiab] OR “relaxation” [tiab] OR “Arteriolar constriction” [tiab] OR “Arteriolar diameter” [tiab] OR “Resting tension” [tiab]

Hyperoxia

"Hyperoxia"[Mesh] OR "Oxygen/therapy"[Mesh] OR "Blood Gas Analysis"[Mesh] OR "Oxygen Inhalation Therapy"[Mesh:noexp] OR "Oxygen/pharmacology"[Mesh] OR "Oxygen/organization and administration"[Mesh] OR "hyperoxia"[tiab] OR "hyperoxaemia"[tiab] OR "hyperoxic"[tiab] OR "hyperoxigenation"[tiab] OR "oxygen exposure"[tiab] OR "oxygen supplementation"[tiab] OR "oxygen administration"[tiab] OR "oxygen sensitivity"[tiab] OR "oxygen induced"[tiab] OR "oxygen tension"[tiab] OR "oxygen sensing"[tiab] OR "oxygen inhalation"[tiab] OR "oxygen insufflation"[tiab] OR "pure oxygen"[tiab] OR "elevated oxygen"[tiab] OR "high blood oxygen"[tiab] OR "high oxygen"[tiab] OR "O2 exposure"[tiab] OR "O2 supplementation"[tiab] OR "O2 administration"[tiab] OR "O2 sensitivity"[tiab] OR "O2 induced"[tiab] OR "O2 tension"[tiab] OR "O2 sensing"[tiab] OR "O2 inhalation"[tiab] OR "O2 insufflation"[tiab] OR "pure O2"[tiab] OR "elevated O2"[tiab] OR "high blood O2"[tiab] OR "high O2"[tiab] OR "100% Oxygen"[tiab] OR "100% O2"[tiab] OR "tissue oxygenation"[tiab] OR “PO2” [tiab] OR “PO(2)”[tiab] OR “PO(2)s”[tiab] OR “PO(2)’s”[tiab] OR “PaO2” [tiab]

Animals

(“animal experimentation”[MeSH Terms] OR “models, animal”[MeSH Terms] OR “invertebrates”[MeSH Terms] OR “Animals”[Mesh:noexp] OR “animal population groups”[MeSH Terms] OR “chordata”[MeSH Terms:noexp] OR “chordata, nonvertebrate”[MeSH Terms] OR “vertebrates”[MeSH Terms:noexp] OR “amphibians”[MeSH Terms] OR “birds”[MeSH Terms] OR “fishes”[MeSH Terms] OR “reptiles”[MeSH Terms] OR “mammals”[MeSH Terms:noexp] OR “primates”[MeSH Terms:noexp] OR “artiodactyla”[MeSH Terms] OR “carnivora”[MeSH Terms] OR “cetacea”[MeSH Terms] OR “chiroptera”[MeSH Terms] OR “elephants”[MeSH Terms] OR “hyraxes”[MeSH Terms] OR “insectivora”[MeSH Terms] OR “lagomorpha”[MeSH Terms] OR “marsupialia”[MeSH Terms] OR “monotremata”[MeSH Terms] OR “perissodactyla”[MeSH Terms] OR “rodentia”[MeSH Terms] OR “scandentia”[MeSH Terms] OR “sirenia”[MeSH Terms] OR “xenarthra”[MeSH Terms] OR “haplorhini”[MeSH Terms:noexp] OR “strepsirhini”[MeSH Terms] OR “platyrrhini”[MeSH Terms] OR “tarsii”[MeSH Terms] OR “catarrhini”[MeSH Terms:noexp] OR “cercopithecidae”[MeSH Terms] OR “hylobatidae”[MeSH Terms] OR “hominidae”[MeSH Terms:noexp] OR “gorilla gorilla”[MeSH Terms] OR “pan paniscus”[MeSH Terms] OR “pan troglodytes”[MeSH Terms] OR “pongo pygmaeus”[MeSH Terms]) OR ((animals[tiab] OR animal[tiab] OR mice[Tiab] OR mus[Tiab] OR mouse[Tiab] OR murine[Tiab] OR woodmouse[tiab] OR rats[Tiab] OR rat[Tiab] OR murinae[Tiab] OR muridae[Tiab] OR cottonrat[tiab] OR cottonrats[tiab] OR hamster[tiab] OR hamsters[tiab] OR cricetinae[tiab] OR rodentia[Tiab] OR rodent[Tiab] OR rodents[Tiab] OR pigs[Tiab] OR pig[Tiab] OR swine[tiab] OR swines[tiab] OR piglets[tiab] OR piglet[tiab] OR boar[tiab] OR boars[tiab] OR “sus scrofa”[tiab] OR

ferrets[tiab] OR ferret[tiab] OR ferrets[tiab] OR polecat[tiab] OR polecats[tiab] OR “mustela putorius”[tiab] OR “guinea pigs”[Tiab] OR “guinea pig”[Tiab] OR cavia[Tiab] OR callithrix[Tiab] OR marmoset[Tiab] OR marmosets[Tiab] OR cebuella[Tiab] OR hapale[Tiab] OR octodon[Tiab] OR chinchilla[Tiab] OR chinchillas[Tiab] OR gerbillinae[Tiab] OR gerbil[Tiab] OR gerbils[Tiab] OR jird[Tiab] OR jirds[Tiab] OR merione[Tiab] OR meriones[Tiab] OR rabbits[Tiab] OR rabbit[Tiab] OR hares[Tiab] OR hare[Tiab] OR diptera[Tiab] OR flies[Tiab] OR fly[Tiab] OR dipteral[Tiab] OR drosphila[Tiab] OR drosophilidae[Tiab] OR cats[Tiab] OR cat[Tiab] OR carus[Tiab] OR felis[Tiab] OR nematoda[Tiab] OR nematode[Tiab] OR nematoda[Tiab] OR nematode[Tiab] OR nematodes[Tiab] OR sipunculida[Tiab] OR dogs[Tiab] OR dog[Tiab] OR canine[Tiab] OR canines[Tiab] OR canis[Tiab] OR sheep[Tiab] OR sheeps[Tiab] OR mouflon[Tiab] OR mouflons[Tiab] OR ovis[Tiab] OR goats[Tiab] OR goat[Tiab] OR goats[Tiab] OR capra[Tiab] OR capras[Tiab] OR rupicapra[Tiab] OR rupicapra[Tiab] OR chamois[Tiab] OR haplorhini[Tiab] OR monkey[Tiab] OR monkeys[Tiab] OR anthropoidea[Tiab] OR anthropoids[Tiab] OR saguinus[Tiab] OR tamarin[Tiab] OR tamarins[Tiab] OR leontopithecus[Tiab] OR hominidae[Tiab] OR ape[Tiab] OR apes[Tiab] OR pan[Tiab] OR paniscus[Tiab] OR “pan paniscus”[Tiab] OR bonobo[Tiab] OR bonobos[Tiab] OR troglodytes[Tiab] OR “pan troglodytes”[Tiab] OR gibbon[Tiab] OR gibbons[Tiab] OR siamang[Tiab] OR siamangs[Tiab] OR nomascus[Tiab] OR symphalangus[Tiab] OR chimpanzee[Tiab] OR chimpanzees[Tiab] OR prosimians[Tiab] OR “bush baby”[Tiab] OR prosimian[Tiab] OR bush babies[Tiab] OR galagos[Tiab] OR galago[Tiab] OR pongidae[Tiab] OR gorilla[Tiab] OR gorilla[Tiab] OR pongo[Tiab] OR pygmaeus[Tiab] OR “pongo pygmaeus”[Tiab] OR orangutans[Tiab] OR pygmaeus[Tiab] OR lemur[Tiab] OR lemurs[Tiab] OR lemuridae[Tiab] OR horse[Tiab] OR horses[Tiab] OR pongo[Tiab] OR equus[Tiab] OR cow[Tiab] OR calf[Tiab] OR bull[Tiab] OR chicken[Tiab] OR chickens[Tiab] OR gallus[Tiab] OR quail[Tiab] OR bird[Tiab] OR birds[Tiab] OR quails[Tiab] OR poultry[Tiab] OR poultries[Tiab] OR fowl[Tiab] OR fowls[Tiab] OR reptile[Tiab] OR reptilia[Tiab] OR reptiles[Tiab] OR snakes[Tiab] OR snake[Tiab] OR lizard[Tiab] OR lizards[Tiab] OR alligator[Tiab] OR alligators[Tiab] OR crocodile[Tiab] OR crocodiles[Tiab] OR turtle[Tiab] OR turtles[Tiab] OR amphibian[Tiab] OR amphibians[Tiab] OR amphibia[Tiab] OR frog[Tiab] OR frogs[Tiab] OR bombina[Tiab] OR salientia[Tiab] OR toad[Tiab] OR toads[Tiab] OR “epidalea calamita”[Tiab] OR salamander[Tiab] OR salamanders[Tiab] OR eel[Tiab] OR eels[Tiab] OR fish[Tiab] OR fishes[Tiab] OR pisces[Tiab] OR catfish[Tiab] OR catfishes[Tiab] OR siluriformes[Tiab] OR arius[Tiab] OR heteropneustes[Tiab] OR sheatfish[Tiab] OR perch[Tiab] OR perches[Tiab] OR percidae[Tiab] OR perca[Tiab] OR trout[Tiab] OR trouts[Tiab] OR char[Tiab] OR chars[Tiab] OR salvelinus[Tiab] OR “fathead minnow”[Tiab] OR minnow[Tiab] OR cyprinidae[Tiab] OR carps[Tiab] OR carp[Tiab] OR zebrafish[Tiab] OR zebrafishes[Tiab] OR goldfish[Tiab] OR goldfishes[Tiab] OR guppy[Tiab] OR guppies[Tiab] OR chub[Tiab] OR chubs[Tiab] OR tinca[Tiab] OR barbels[Tiab] OR barbus[Tiab] OR pimephales[Tiab] OR promelas[Tiab] OR “poecilia reticulata”[Tiab] OR mullet[Tiab] OR mullets[Tiab] OR eel[Tiab] OR eels[Tiab] OR seahorse[Tiab] OR seahorses[Tiab] OR mugil curema[Tiab] OR atlantic cod[Tiab] OR shark[Tiab] OR sharks[Tiab] OR catshark[Tiab] OR anguilla[Tiab] OR salmonid[Tiab] OR salmonids[Tiab] OR whitefish[Tiab] OR whitefishes[Tiab] OR salmon[Tiab] OR salmons[Tiab] OR sole[Tiab] OR solea[Tiab] OR “sea lamprey”[Tiab] OR lamprey[Tiab] OR lampreys[Tiab] OR pumpkinseed[Tiab] OR sunfish[Tiab] OR sunfishes[Tiab] OR tilapia[Tiab] OR tilapias[Tiab] OR turbot[Tiab] OR turbots[Tiab] OR flatfish[Tiab] OR flatfishes[Tiab] OR sciuridae[Tiab] OR squirrel[Tiab] OR squirrels[Tiab] OR chipmunk[Tiab] OR chipmunks[Tiab] OR suslik[Tiab] OR susliks[Tiab] OR vole[Tiab] OR voles[Tiab] OR lemming[Tiab] OR lemmings[Tiab] OR muskrat[Tiab] OR muskrats[Tiab] OR lemmus[Tiab] OR otter[Tiab] OR otters[Tiab] OR marten[Tiab] OR martens[Tiab] OR martes[Tiab] OR weasel[Tiab] OR badger[Tiab] OR ermine[Tiab] OR mink[Tiab] OR sable[Tiab] OR sables[Tiab] OR badgers[Tiab] OR gulo[Tiab] OR gulos[Tiab] OR wolverine[Tiab] OR wolverines[Tiab] OR minks[Tiab] OR mustela[Tiab] OR llama[Tiab] OR llamas[Tiab] OR alpaca[Tiab] OR alpacas[Tiab] OR camelid[Tiab] OR camelids[Tiab] OR guanaco[Tiab] OR guanacos[Tiab] OR chiroptera[Tiab] OR chiropteras[Tiab] OR bat[Tiab] OR bats[Tiab] OR fox[Tiab] OR foxes[Tiab] OR iguana[Tiab] OR iguanas[Tiab] OR xenopus laevis[Tiab] OR parakeet[Tiab] OR parakeets[Tiab] OR parrot[Tiab] OR parrots[Tiab] OR donkey[Tiab] OR donkeys[Tiab] OR mule[Tiab] OR mules[Tiab] OR zebra[Tiab] OR zebras[Tiab] OR shrew[Tiab] OR shrews[Tiab] OR bison[Tiab] OR bisons[Tiab] OR buffalo[Tiab] OR buffaloes[Tiab] OR deer[Tiab] OR deers[Tiab] OR bear[Tiab] OR bears[Tiab] OR panda[Tiab] OR pandas[Tiab] OR “wild hog”[Tiab] OR “wild boar”[Tiab] OR fitchew[Tiab] OR fitch[Tiab] OR beaver[Tiab] OR beavers[Tiab] OR jerboa[Tiab] OR jerboas[Tiab] OR capybara[Tiab] OR capybaras[Tiab] OR minks[Tiab] OR canine [tiab] OR bovine [tiab] OR porcine [tiab] OR hog [tiab] OR hogs [tiab]) NOT medline[sb])

EMBASE query

Vasoconstriction/vasodilatation

‘vasoconstriction’/exp OR ‘blood vessel tone’/exp OR ‘blood vessel diameter’/exp OR ‘vasodilatation’/exp OR ‘artery diameter’/exp OR ‘vasoconstriction’:ti,ab OR ‘vasoconstrictions’:ti,ab OR ‘vasoconstrictor’:ti,ab OR ‘vasoconstrictors’:ti,ab OR ‘vasopressor’:ti,ab OR ‘vasoactive agonist’:ti,ab OR ‘vasoactive agonists’:ti,ab OR ‘vasopressors’:ti,ab OR ‘vasomotor system’:ti,ab OR ‘artery constriction’:ti,ab OR ‘vessel constriction’:ti,ab OR ‘vasoconstrictive’:ti,ab OR ‘vasoconstricting’:ti,ab OR ‘vasoconstricted’:ti,ab OR ‘vasodilation’:ti,ab OR ‘vasodilatation’:ti,ab OR ‘vasodilatating’:ti,ab OR ‘vasodilating’:ti,ab OR ‘vasodilative’:ti,ab OR ‘vasodilatative’:ti,ab OR ‘artery dilation’:ti,ab OR ‘vessel dilation’:ti,ab OR ‘artery dilatation’:ti,ab OR ‘vessel dilatation’:ti,ab OR ‘vasodilator’:ti,ab OR ‘vasodilators’:ti,ab OR ‘vasorelaxation’:ti,ab OR ‘vascular endothelium dependent relaxation’:ti,ab OR ‘endothelium dependent relaxation’:ti,ab OR ‘vascular endothelium-dependent relaxation’:ti,ab OR ‘endothelium-dependent-relaxation’:ti,ab OR ‘vasodilated’:ti,ab OR ‘vasoactive agent’:ti,ab OR ‘vasoactive drug’:ti,ab OR ‘vasoactive drugs’:ti,ab OR ‘dilation’:ti,ab OR ‘dilatation’:ti,ab OR ‘contraction’:ti,ab OR ‘relaxation’:ti,ab OR ‘Arteriolar constriction’:ti,ab OR ‘Arteriolar diameter’:ti,ab OR ‘resting tension’:ti,ab

Hyperoxia

‘Hyperoxia’/exp OR ‘Oxygen therapy’/exp OR ‘hyperoxia’:ti,ab OR ‘hyperoxaemia’:ti,ab OR ‘hyperoxic’:ti,ab OR ‘hyperoxygenation’:ti,ab OR ‘oxygen exposure’:ti,ab OR ‘oxygen supplementation’:ti,ab OR‘oxygen administration’:ti,ab OR ‘oxygen sensitivity’:ti,ab OR ‘oxygen induced’:ti,ab OR ‘oxygen tension’:ti,ab OR ‘oxygen sensing’:ti,ab OR ‘oxygen inhalation’:ti,ab OR ‘oxygen insufflation’:ti,ab OR ‘pure oxygen’:ti,ab OR ‘elevated oxygen’:ti,ab OR ‘high blood oxygen’:ti,ab OR ‘high oxygen’:ti,ab OR ‘O2 exposure’:ti,ab OR ‘O2 supplementation’:ti,ab OR ‘O2 administration’:ti,ab OR ‘O2 sensitivity’:ti,ab OR ‘O2 induced’:ti,ab OR ‘O2 tension’:ti,ab OR ‘O2 sensing’:ti,ab OR ‘O2 inhalation’:ti,ab OR ‘O2 insufflation’:ti,ab OR ‘pure O2’:ti,ab OR ‘elevated O2’:ti,ab OR ‘high blood O2’:ti,ab OR ‘high O2’:ti,ab OR ‘100% Oxygen’:ti,ab OR ‘100% O2’:ti,ab OR ‘tissue oxygenation’:ti,ab OR ‘PO2’:ti,ab OR ‘PO(2)’:ti,ab OR ‘PO(2)s’:ti,ab OR ‘PO(2)/s’:ti,ab OR ‘PaO2’:ti,ab

Animals

‘animal experiment’/exp OR ‘animal model’/exp OR ‘experimental animal’/exp OR ‘transgenic animal’/exp OR ‘male animal’/exp OR ‘female animal’/exp OR ‘juvenile animal’/exp OR ‘animal’/exp OR ‘chordata’/exp OR ‘vertebrate’/exp OR ‘tetrapod’/exp OR ‘fish’/exp OR ‘amniote’/exp OR ‘amphibia’/exp OR ‘mammal’/exp OR ‘reptile’/exp OR ‘sauropsid’/exp OR ‘therian’/exp OR ‘monotremate’/exp OR ‘placental mammals’/exp OR ‘marsupial’/exp OR ‘Euarchontoglires’/exp OR ‘Afrotheria’/exp OR ‘Boreoeutheria’/exp OR ‘Laurasiatheria’/exp OR ‘Xenarthra’/exp OR ‘primate’/exp OR ‘Dermoptera’/exp OR ‘Glires’/exp OR ‘Scandentia’/exp OR ‘Haplorhini’/exp OR ‘prosimian’/exp OR ‘simian’/exp OR ‘tarsiiform’/exp OR ‘Catarrhini’/exp OR ‘Platyrrhini’/exp OR ‘ape’/exp OR ‘Cercopithecidae’/exp OR ‘hominid’/exp OR ‘hylobatidae’/exp OR ‘chimpanzee’/exp OR ‘gorilla’/exp OR ‘orang utan’/exp OR (‘animal’ OR ‘animals’ OR ‘pisces’ OR ‘fish’ OR ‘fishes’ OR ‘catfish’ OR ‘catfishes’ OR ‘sheatfish’ OR ‘silurus’ OR ‘arius’ OR ‘heteropneustes’ OR ‘clarias’ OR ‘gariepinus’ OR ‘fathead minnow’ OR ‘fathead minnows’ OR ‘pimephales’ OR ‘promelas’ OR ‘cichlidae’ OR ‘trout’ OR ‘trouts’ OR ‘char’ OR ‘chars’ OR ‘salvelinus’ OR ‘salmo’ OR ‘oncorhynchus’ OR ‘guppy’ OR ‘guppies’ OR ‘millionfish’ OR ‘poecilia’ OR ‘goldfish’ OR ‘goldfishes’ OR ‘carassius’ OR ‘auratus’ OR ‘mullet’ OR ‘mullets’ OR ‘mugil’ OR ‘curema’ OR ‘shark’ OR ‘sharks’ OR ‘cod’ OR ‘cods’ OR ‘gadus’ OR ‘morhua’ OR ‘carp’ OR ‘carps’ OR ‘cyprinus’ OR ‘carpio’ OR ‘killifish’ OR ‘eel’ OR ‘eels’ OR ‘anguilla’ OR ‘zander’ OR ‘sander’ OR ‘lucioperca’ OR ‘stizostedion’ OR ‘turbot’ OR ‘turbots’ OR ‘psetta’ OR ‘flatfish’ OR ‘flatfishes’ OR ‘plaice’ OR ‘pleuronectes’ OR ‘platessa’ OR ‘tilapia’ OR ‘tilapias’ OR ‘oreochromis’ OR ‘sarotherodon’ OR ‘common sole’ OR ‘dover sole’ OR ‘solea’ OR ‘zebrafish’ OR ‘zebrafishes’ OR ‘danio’ OR ‘rerio’ OR ‘seabass’ OR ‘dicentrarchus’ OR ‘labrax’ OR ‘morone’ OR ‘lamprey’ OR ‘lampreys’ OR ‘petromyzon’ OR ‘pumpkinseed’ OR ‘pumpkinseeds’ OR ‘lepomis’ OR ‘gibbosus ‘ OR ‘herring’ OR ‘clupea’ OR ‘harengus’ OR ‘amphibia’ OR ‘amphibian’ OR ‘amphibians’ OR ‘anura’ OR ‘salientia’ OR ‘frog’ OR ‘frogs’ OR ‘rana’

OR ‘toad’ OR ‘toads’ OR ‘bufo’ OR ‘xenopus’ OR ‘laevis’ OR ‘bombina’ OR ‘epidalea’ OR ‘calamita’ OR ‘salamander’ OR ‘salamanders’ OR ‘newt’ OR ‘newts’ OR ‘triturus’ OR ‘reptilia’ OR ‘reptile’ OR ‘reptiles’ OR ‘bearded dragon’ OR ‘pogona’ OR ‘vitticeps’ OR ‘iguana’ OR ‘iguanas’ OR ‘lizard’ OR ‘lizards’ OR ‘anguis fragilis’ OR ‘turtle’ OR ‘turtles’ OR ‘snakes’ OR ‘snake’ OR ‘aves’ OR ‘bird’ OR ‘birds’ OR ‘quail’ OR ‘quails’ OR ‘coturnix’ OR ‘bobwhite’ OR ‘colinus’ OR ‘virginianus’ OR ‘poultry’ OR ‘poultries’ OR ‘fowl’ OR ‘fowls’ OR ‘chicken’ OR ‘chickens’ OR ‘gallus’ OR ‘zebra finch’ OR ‘taeniopygia’ OR ‘guttata’ OR ‘canary’ OR ‘canaries’ OR ‘serinus’ OR ‘canaria’ OR ‘parakeet’ OR ‘parakeets’ OR ‘grasskeet’ OR ‘parrot’ OR ‘parrots’ OR ‘psittacine’ OR ‘psittacines’ OR ‘shelduck’ OR ‘tadorna’ OR ‘goose’ OR ‘geese’ OR ‘branta’ OR ‘leucopsis’ OR ‘woodlark’ OR ‘lullula’ OR ‘flycatcher’ OR ‘ficedula’ OR ‘hypoleuca’ OR ‘dove’ OR ‘doves’ OR ‘geopelia’ OR ‘cuneata’ OR ‘duck’ OR ‘ducks’ OR ‘greylag’ OR ‘graylag’ OR ‘anser’ OR ‘harrier’ OR ‘circus pygargus’ OR ‘red knot’ OR ‘great knot’ OR ‘calidris’ OR ‘canutus’ OR ‘godwit’ OR ‘limosa’ OR ‘lapponica’ OR ‘meleagris’ OR ‘gallopavo’ OR ‘jackdaw’ OR ‘corvus’ OR ‘monedula’ OR ‘ruff’ OR ‘philomachus’ OR ‘pugnax’ OR ‘lapwing’ OR ‘peewit’ OR ‘plover’ OR ‘vanellus’ OR ‘swan’ OR ‘cygnus’ OR ‘columbianus’ OR ‘bewickii’ OR ‘gull’ OR ‘chroicocephalus’ OR ‘ridibundus’ OR ‘albifrons’ OR ‘great tit’ OR ‘parus’ OR ‘aythya’ OR ‘fuligula’ OR ‘streptopelia’ OR ‘risoria’ OR ‘spoonbill’ OR ‘platalea’ OR ‘leucorodia’ OR ‘blackbird’ OR ‘turdus’ OR ‘merula’ OR ‘blue tit’ OR ‘cyanistes’ OR ‘pigeon’ OR ‘pigeons’ OR ‘columba’ OR ‘pintail’ OR ‘anas’ OR ‘starling’ OR ‘sturnus’ OR ‘owl’ OR ‘athene noctua’ OR ‘pochard’ OR ‘ferina’ OR ‘cockatiel’ OR ‘nymphicus’ OR ‘hollandicus’ OR ‘skylark’ OR ‘alauda’ OR ‘tern’ OR ‘sterna’ OR ‘teal’ OR ‘crecca’ OR ‘oystercatcher’ OR ‘haematopus’ OR ‘ostralegus’ OR ‘shrew’ OR ‘shrews’ OR ‘sorex’ OR ‘araneus’ OR ‘crocidura’ OR ‘russula’ OR ‘european mole’ OR ‘talpa’ OR ‘chiroptera’ OR ‘bat’ OR ‘bats’ OR ‘eptesicus’ OR ‘serotinus’ OR ‘myotis’ OR ‘dasycneme’ OR ‘daubentonii’ OR ‘pipistrelle’ OR ‘pipistrellus’ OR ‘cat’ OR ‘cats’ OR ‘felis’ OR ‘catus’ OR ‘feline’ OR ‘dog’ OR ‘dogs’ OR ‘canis’ OR ‘canine’ OR ‘canines’ OR ‘otter’ OR ‘otters’ OR ‘lutra’ OR ‘badger’ OR ‘badgers’ OR ‘meles’ OR ‘fitchew’ OR ‘fitch’ OR ‘foumart’ OR ‘foulmart’ OR ‘ferrets’ OR ‘ferret’ OR ‘polecat’ OR ‘polecats’ OR ‘mustela’ OR ‘putorius’ OR ‘weasel’ OR ‘weasels’ OR ‘fox’ OR ‘foxes’ OR ‘vulpes’ OR ‘common seal’ OR ‘phoca’ OR ‘vitulina’ OR ‘grey seal’ OR ‘halichoerus’ OR ‘horse’ OR ‘horses’ OR ‘equus’ OR ‘equine’ OR ‘equidae’ OR ‘donkey’ OR ‘donkeys’ OR ‘mule’ OR ‘mules’ OR ‘pig’ OR ‘pigs’ OR ‘swine’ OR ‘swines’ OR ‘hog’ OR ‘hogs’ OR ‘boar’ OR ‘boars’ OR ‘porcine’ OR ‘piglet’ OR ‘piglets’ OR ‘sus’ OR ‘scrofa’ OR ‘llama’ OR ‘llamas’ OR ‘lama’ OR ‘glama’ OR ‘deer’ OR ‘deers’ OR ‘cervus’ OR ‘elaphus’ OR ‘cow’ OR ‘cows’ OR ‘bos taurus’ OR ‘bos indicus’ OR ‘bovine’ OR ‘bull’ OR ‘bulls’ OR ‘cattle’ OR ‘bison’ OR ‘bisons’ OR ‘sheep’ OR ‘sheeps’ OR ‘ovis aries’ OR ‘ovine’ OR ‘lamb’ OR ‘lambs’ OR ‘mouflon’ OR ‘mouflons’ OR ‘goat’ OR ‘goats’ OR ‘capra’ OR ‘caprine’ OR ‘chamois’ OR ‘rupicapra’ OR ‘leporidae’ OR ‘lagomorpha’ OR ‘lagomorph’ OR ‘rabbit ‘ OR ‘rabbits’ OR ‘oryctolagus’ OR ‘cuniculus’ OR ‘laprine’ OR ‘hares’ OR ‘lepus’ OR ‘rodentia’ OR ‘rodent’ OR ‘rodents’ OR ‘murinae’ OR ‘mouse’ OR ‘mice’ OR ‘mus’ OR ‘musculus’ OR ‘murine’ OR ‘woodmouse’ OR ‘apodemus’ OR ‘rat’ OR ‘rats’ OR ‘rattus’ OR ‘norvegicus’ OR ‘guinea pig’ OR ‘guinea pigs’ OR ‘cavia’ OR ‘porcellus’ OR ‘hamster’ OR ‘hamsters’ OR ‘mesocricetus’ OR ‘cricetulus’ OR ‘cricetus’ OR ‘gerbil’ OR ‘gerbils’ OR ‘jird’ OR ‘jirds’ OR ‘meriones’ OR ‘unguiculatus’ OR ‘jerboa’ OR ‘jerboas’ OR ‘jaculus’ OR ‘chinchilla’ OR ‘chinchillas’ OR ‘beaver’ OR ‘beavers’ OR ‘castor fiber’ OR ‘castor canadensis’ OR ‘sciuridae’ OR ‘squirrel’ OR ‘squirrels’ OR ‘sciurus’ OR ‘chipmunk’ OR ‘chipmunks’ OR ‘marmot’ OR ‘marmots’ OR ‘marmota’ OR ‘suslik’ OR ‘susliks’ OR ‘spermophilus’ OR ‘cynomys’ OR ‘cottonrat’ OR ‘cottonrats’ OR ‘sigmodon’ OR ‘vole’ OR ‘voles’ OR ‘microtus’ OR ‘myodes’ OR ‘glareolus’ OR ‘primate’ OR ‘primates’ OR ‘prosimian’ OR ‘prosimians’ OR ‘lemur’ OR ‘lemurs’ OR ‘lemuridae’ OR ‘loris’ OR ‘bush baby’ OR ‘bush babies’ OR ‘bushbaby’ OR ‘bushbabies’ OR ‘galago’ OR ‘galagos’ OR ‘anthropoidea’ OR ‘anthropoids’ OR ‘simian’ OR ‘simians’ OR ‘monkey’ OR ‘monkeys’ OR ‘marmoset’ OR ‘marmosets’ OR ‘callithrix’ OR ‘cebuella’ OR ‘tamarin’ OR ‘tamarins’ OR ‘saguinus’ OR ‘leontopithecus’ OR ‘squirrel monkey’ OR ‘squirrel monkeys’ OR ‘saimiri’ OR ‘night monkey’ OR ‘night monkeys’ OR ‘owl monkey’ OR ‘owl monkeys’ OR ‘douroucoulis’ OR ‘aotus’ OR ‘spider monkey’ OR ‘spider monkeys’ OR ‘ateles’ OR ‘baboon’ OR ‘baboons’ OR ‘papio’ OR ‘rhesus monkey’ OR ‘macaque’ OR ‘macaca’ OR ‘mulatta’ OR ‘cynomolgus’ OR ‘fascicularis’ OR ‘green monkey’ OR ‘green monkeys’ OR ‘chlorocebus’ OR ‘vervet’ OR ‘vervets’ OR ‘pygerythrus’ OR ‘hominoidea’ OR ‘ape’ OR ‘apes’ OR ‘hylobatidae’ OR ‘gibbon’ OR ‘gibbons’ OR ‘siamang’ OR ‘siamangs’ OR ‘nomascus’ OR ‘symphalangus’ OR ‘hominidae’ OR ‘orangutan’ OR ‘orangutans’ OR ‘pongo’ OR ‘chimpanzee’ OR ‘chimpanzees’ OR ‘pan troglodytes’ OR ‘bonobo’ OR ‘bonobos’ OR ‘pan paniscus’ OR ‘gorilla’ OR ‘gorillas’ OR ‘troglodytes’ OR ‘canine’ OR ‘bovine’ OR ‘porcine’ OR ‘hog’ OR ‘hogs’):ti,ab
