# Supplementary material for: Effects of hyperoxia on vascular tone in animal models: systematic review and meta-analysis
Source: Crit Care. 2018 Aug 4;22:189. doi: 10.1186/s13054-018-2123-9 (PMC6091089; doi:10.1186/s13054-018-2123-9)
Supplement: Supplementary file 2 — Quality checklist. Modified quality checklist used to assess the quality of the included studies. (PDF 14 kb) [file 13054_2018_2123_MOESM2_ESM.pdf]

Supplemental file 2 – Quality checklist and assessment

**Component**	**Comments/Clarification**
1. Were the assessors/data analysts blinded?	Yes: Blinding was performed and adequately described.<br>No: Blinding is not mentioned and/or experiments were always performed in the same order or the process was insufficiently described
2. Was the preparation checked for stability and quality?	Yes: The study included an endothelium/smooth muscle check or similar and investigated the stability of the preparation over time (if applicable)<br>No: Either checks were not described, or checks were performed, but its consequences were not defined
3. Did the authors verify the intervention?	Yes: PO_2s or P_aO_2s were measured and reported<br>No: Neither were measured or reported
4. Did the authors verify temperatures, pH and CO_2?	Yes: Target values were provided and measured for all three properties<br>No: One or more values were not reported<br>Unclear: Target values were reported, but it was unclear whether or how they were measured
5. Was there no selective outcome reporting?	Yes: There was no indication for selective outcome reporting<br>No: An effect was described, but no data was shown
6. Were the animal characteristics properly described?	Yes: The authors mentioned species, age/weight, gender and the number of animals used<br>No: The authors did not describe one or more of the above characteristics
7. Were the vessels properly described?	Yes: The method of artery selection (e.g. order, standardized location), number of vessels per animal used and its diameter were reported<br>No: The authors did not describe the above characteristics
8. Were group sized based on a power analysis?	Yes: A proper power analysis was described<br>No: A power analysis was not described or mentioned
9. Is the study published in a peer reviewed journal?	Yes/No
10. Was the study approved by an ethical board?	Yes/No
11. Is there a conflict of interest statement?	Yes/No
