# Supplementary figures and images for: Effects of hyperoxia on vascular tone in animal models: systematic review and meta-analysis
Source: Crit Care. 2018 Aug 4;22:189. doi: 10.1186/s13054-018-2123-9 (PMC6091089; doi:10.1186/s13054-018-2123-9)

**Supplemental File 4 – Funnel plots**

**Funnel plots of In vivo studies**

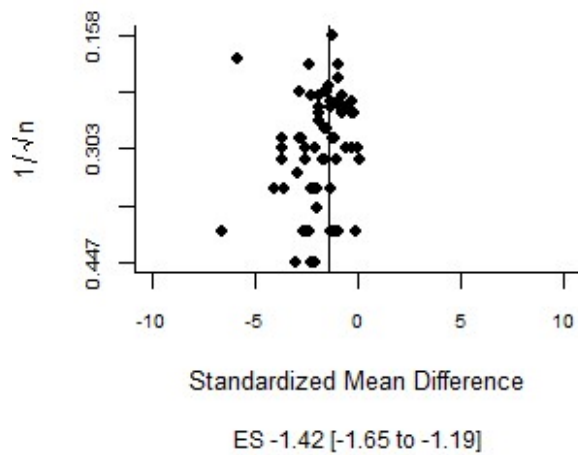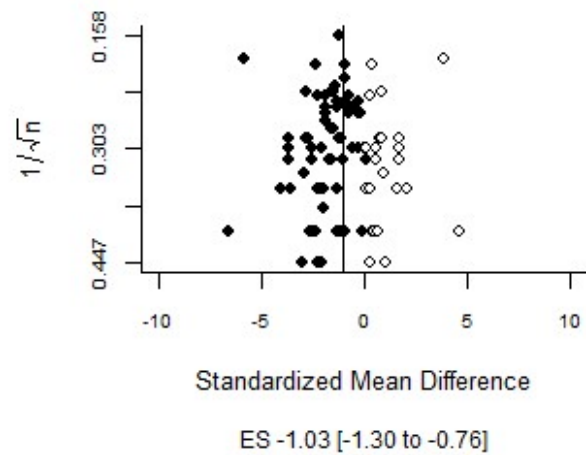

**Funnel plots of ex vivo studies**

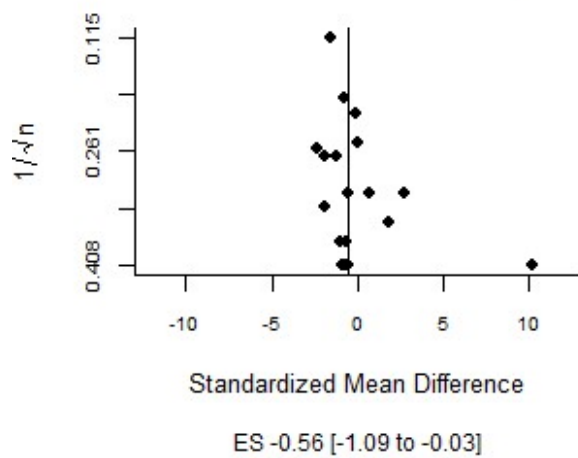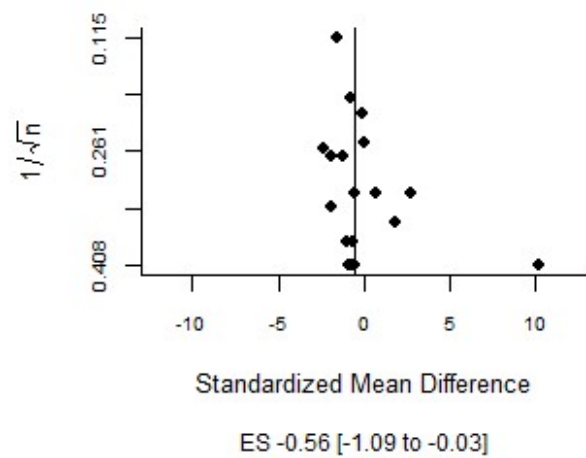

Supplement: Supplementary file 3 — Funnel plots. Results of the publication bias assessment in the form of funnel plots. (PDF 344 kb) [file 13054_2018_2123_MOESM3_ESM.pdf]
