# Supplementary material for: Effects of hyperoxia on vascular tone in animal models: systematic review and meta-analysis
Source: Crit Care. 2018 Aug 4;22:189. doi: 10.1186/s13054-018-2123-9 (PMC6091089; doi:10.1186/s13054-018-2123-9)
Supplement: Supplementary file 4 — Sensitivity analysis. Results of the sensitivity analysis for the correlation coefficients. (PDF 232 kb) [file 13054_2018_2123_MOESM4_ESM.pdf]

Supplemental file 3 – Sensitivity analysis

Meta-analysis

The table below shows the impact of a lower and higher correlation coefficient on the overall effect size for in-vivo and isolated artery studies. A conservative correlation of 0.7 was used in the analysis presented in the manuscript.

Correlation	*In vivo* studies		*Ex vivo* studies	
	SMD	95%CI	SMD	95%CI
0.5	-1.35	[-1.56 to -1.13]	-0.50	[-1.01 to 0.01]
0.7 (used in analysis)	-1.42	[-1.64 to -1.20]	-0.56	[-1.09 to -0.03]
0.9	-1.58	[-1.83 to -1.33]	-0.69	[-1.30 to -0.09]
